# Quantifying the relationship between gardening and health and well-being in the UK: a survey during the covid-19 pandemic

**DOI:** 10.1186/s12889-024-18249-8

**Published:** 2024-03-14

**Authors:** Boglarka Z. Gulyas, Samantha J. Caton, Jill L. Edmondson

**Affiliations:** 1 Plants, Photosynthesis and Soil, School of Biosciences, University of Sheffield, S10 2TN Sheffield, UK; 2 Sheffield Centre for Health and Related Research (SCHARR), School of Medicine and Population Health, University of Sheffield, S10 2TN Sheffield, UK

**Keywords:** Gardening, Urban horticulture, Fruit and vegetable consumption, Mental well-being, Self-rated health, COVID-19

## Abstract

**Background:**

Rates of non-communicable diseases, including cardiovascular disease and type 2 diabetes, and mental health problems, such as anxiety and depression, are high and rising in the urbanising world. Gardening could improve both mental and physical health and help prevent a range of conditions by increasing fruit and vegetable (F&V) consumption, promoting physical activity, and reducing stress. However, good quality quantitative research in the area is scarce, and our understanding of the role of allotments and home gardens, and the effects of the level of engagement in gardening and involvement with food production has thus far been limited.

**Methods:**

We quantitatively assess the relationship between home and allotment gardening and various indicators and predictors of health and well-being using an online survey of gardeners (*n* = 203) and non-gardeners (*n* = 71) in the UK. The survey was composed of multiple validated questionnaires (including the Short Form Food Frequency Questionnaire (SFFFQ), the Warwick-Edinburgh Mental Wellbeing Scale (WEMWBS), the Physical Health Questionnaire (PHQ) and the Self-Rated Health question (SRH)) and self-defined questions relating to participants’ involvement with gardening and food production, and relevant demographic and lifestyle factors. Data were analysed using a series of hierarchical logistic and multiple linear regression models adjusting for socio-demographic variables.

**Results:**

After adjusting for relevant socio-demographic factors, gardening related variables were associated with better self-rated health, higher mental well-being, increased F&V consumption. Higher F&V intake was in turn also associated with better self-rated health and decreased odds of obesity. Thus, gardening had a positive association with four different aspects of health and well-being, directly or indirectly via increased F&V consumption.

**Conclusions:**

Our results suggest that gardening in UK allotments and domestic gardens may promote different aspects of health and well-being via multiple mechanisms. Improving access to growing space and promoting regular gardening could provide a range of benefits to public health. More research on how socio-economic factors influence the health and well-being benefits of gardening will help policymakers devise strategies to maximise these benefits.

**Supplementary Information:**

The online version contains supplementary material available at 10.1186/s12889-024-18249-8.

## Background

Health and well-being are key determinants of both individuals’ quality of life and of social and economic development [1, 2]. A growing number of people are affected by non-communicable diseases (NCDs), including diabetes, heart disease, stroke and cancer, which are the leading cause of death globally [3, 4] and in the UK [5]. However, while various genetic, socio-demographic and environmental factors increase the risk of developing NCDs, the recent rise in their incidence can be largely attributed to modifiable lifestyle factors, which makes most NCDs preventable [6]. Smoking, unhealthy diets, including low fruit and vegetable (F&V) intake [7, 8] and high processed food [9, 10] and meat [11, 12] consumption, physical inactivity [13, 14] and associated hypertension and obesity [15, 16] are among the main preventable causes of NCDs. In the UK, nearly two thirds of the adult population are overweight or obese [17], and food-related ill health is estimated to cost the National Health Service £6 billion each year [18]. One of the main preventable causes of NCDs in the UK is low F&V consumption—over two thirds of the population do not meet the recommended ‘5-a-day’, which contributes to around 18,000 premature deaths annually [19]. In addition, over a third of adults are not active enough for good health, which is associated with 1 in 6 deaths and an annual healthcare cost of £1.2 billion, and rates of insufficient physical activity are growing [20, 21]. Disruptions caused by the recent covid-19 pandemic had further negative impacts on the eating habits and physical activity levels of many people [22].

As well as physical health, poor mental well-being is a major factor reducing quality of life, in the UK and worldwide [23, 24]. Based on a 2007 survey, 1 in 4 people in England experienced a mental health problem, such as depression or anxiety, each year [25], and mental illness is the second-largest source of burden of disease in the country [26]. To exacerbate the problem, the wide-ranging impacts of the covid-19 pandemic brought about a nationwide decline in mental health and a widening of pre-existing inequalities [27–29]. Similar trends could be observed in other parts of the world [30, 31]. Often devastating on their own, mental health problems also increase the likelihood of unhealthy behaviours and preventable physical health conditions [32–34]. On the other hand, having poor physical health increases the risk of mental health issues, creating a vicious cycle [35]. But the connection between mental health status and modifiable environmental and behavioural factors also creates opportunities for improvement [36, 37].

Lifestyle changes, including increasing physical activity, improving diet quality, and engaging in activities that reduce stress and provide a sense of well-being, can have positive effects on both mental and physical health. As a form of outdoor exercise and an opportunity to relax and connect with nature, ourselves and others, gardening has been used as a therapeutic tool in different settings [38, 39], such as care homes and hospitals, and its potential benefits for health and well-being have been increasingly studied in recent decades. Spending time outdoors, especially in natural environments, has been linked to psychological benefits [40–43], and research suggests that gardening is associated with better mental well-being [44–52], increased physical activity [49, 53], higher F&V intake [54–61], decreased odds of developing overweight and obesity [56, 58, 62–64], and improved strength and flexibility in older adults [51]. Thus, gardening, particularly F&V growing, may offer a way to simultaneously promote health and well-being through a range of pathways.

However, recent systematic reviews have found that good quality quantitative research on the health and well-being benefits of gardening that use validated tools are still relatively scarce, especially in non-institutionalised settings, and most of these have focused on community gardens in the USA [46, 56, 65–68]. Much less is known about the health-promoting potential of fruit and vegetable gardening in domestic gardens and allotments, typical sites of gardening in Europe, and how this may be modulated by the level of engagement. A key challenge in studying this is that health and well-being are multifaceted concepts that are not straightforward to assess, and are influenced by a multitude of interrelated socio-economic, environmental, lifestyle and genetic factors [69, 70]. For example, income, neighbourhood deprivation and educational attainment, as well as age, gender and ethnicity, are correlated with many health outcomes, including the incidence of different diseases, various measures of physical and mental well-being, and certain risk factors to health, such as smoking, alcohol consumption, diet quality, physical activity level and body mass index (BMI) [6]. Therefore, to meaningfully assess the health-promoting effects of gardening, potential confounders must also be considered.

Gardening on allotments (plots of land rented out to individuals for growing fruits and vegetables) and in domestic gardens is a popular recreational activity in the UK that has enjoyed increasing interest in the past 20 years, which grew further during the lockdowns that followed the outbreak of the covid-19 pandemic, motivated by a need to spend time in isolation meaningfully as well as concerns over food shortages [71–74]. Qualitative studies have found that home gardens and allotments can hold important emotional, psychological, and spiritual values for people [75, 76], which may have been a key factor contributing to their rising popularity during a time of great distress and uncertainty. One quantitative study also found that older allotment gardeners in the UK had lower perceived stress levels than similar age participants of indoor exercise classes [47], suggesting a potential role of allotments in improving well-being. Similarly, a pre-pandemic analysis of a representative survey of the English population revealed an association between access to a private garden and better evaluative well-being [77], while another, more recent, study has found that frequent home gardeners had higher mental well-being and lower stress scores and were more physically active than occasional or never-gardeners [78]. Furthermore, research has demonstrated that F&V consumption in UK food-grower households is 70% higher compared to the national average [79]. Although these findings are promising, there is still much we do not know about the contribution of gardening to better health and well-being in the country, such as the amount of time spent gardening that is required to bring about certain benefits, the role of the level of engagement with food production, and potential differences between the benefits of home- and allotment gardening.

The aim of this research was to quantitatively assess the relationship between gardening and health and well-being in the UK to increase our understanding of the ways in which it can exert its beneficial effects. Specifically, we looked at whether different gardening related variables, namely the amount of time spent gardening in a typical week, self-reported amount of food produced, and having an allotment, were associated with better self-rated general health, higher mental well-being, fewer physical health complaints, or certain predictors of health and well-being, namely obesity, diet quality (in particular, F&V intake and meat avoidance), and physical activity level. Better understanding the associations between these variables will help identify the mechanisms by which gardening could improve different aspects of health and well-being, and provide a foundation for efforts aimed at improving public health and well-being.

## Methods

### Participants

Data was collected from adults living in the UK, including both regular gardeners and non-gardeners. Participants were recruited by means of social media (i.e. Facebook, Twitter and email newsletter) and word of mouth in the gardening and food-growing community via the network of the ongoing MYHarvest citizen science project (myharvest.org.uk) [80], in collaboration with the National Allotment Society and the Royal Horticultural Society, and via email through the University of Sheffield’s staff and student volunteer lists. The project was granted ethical approval by the Department of Animal and Plant Sciences of The University of Sheffield (project ref. 041219).

### Materials

We used an online survey composed of validated questionnaires and self-defined questions administered to participants via the Qualtrics platform. Data collection ran between 29th July and 30th November 2020. The survey collected data on various aspects of participants’ health and well-being, their involvement with gardening, and relevant demographic and lifestyle factors (see Additional File 2 for the list of questions).

### Health and well-being measures

#### General health

General health was assessed with the widely used Self-Rated Health (SRH) question (‘*In general, how would you rate your health in the past year? Excellent / Very good / Good / Fair / Poor*’) [81]. The SRH was chosen as a simple yet valid and efficient measure of physical and mental health and predictor of mortality [82, 83].

#### Physical health

The Physical Health Questionnaire (PHQ) by Schat & Kelloway 2005 [84] (a modified version of Spence et al.’s (1987) measure of health [85]) was used as a measure of physical well-being based on the frequency of somatic symptoms experienced by participants, including sleep disturbances, headaches, respiratory illness, and gastrointestinal problems, during the previous month. The PHQ consists of 14 items measured on a seven-point Likert scale. PHQ scores were calculated by totalling responses across all items (with item four reverse scored). Total scores can range from 14 to 98, with higher scores reflecting more frequent physical complaints thus indicating poorer health.

#### Mental well-being

Mental well-being was measured using the Warwick-Edinburgh Mental Well-being Scale (WEMWBS) [86], a widely used tool developed for the measurement of mental well-being in the general population and the evaluation of projects and policies aimed at improving mental well-being. The WEMWBS focuses on feelings and functioning aspects of positive mental well-being in the past two weeks, and consists of 14 positively scored items measured on a 5-point Likert scale. WEMWBS scores were calculated by adding up points for all 14 items, with total scores thus ranging from 14 to 70. Scores less than 43 are considered to indicate *low*, 43 to 60 *moderate*, and above 60 *high* levels of mental well-being.

#### Body Mass Index (BMI)

Body Mass Index (BMI) was calculated from reported height and weight of participants using the formula *weight(kg)/height(m)*^*2*^, and BMI categories were assigned based on these values (BMI < 18.5– *underweight*, 18.5 to 24.9– *healthy weight*, 25 to 29.9– *overweight*, 30 to 39.9– *obese*) [87]. Having obesity was considered as an indicator of increased health risk.

#### Diet quality

Diet quality was intended to be measured using the Short Form Food Frequency Questionnaire (SFFFQ) by Cleghorn et al. 2016 [88], which assesses fruit, vegetable, fat, oily fish and non-milk extrinsic sugar consumption during a typical week over the previous month and allows the calculation of a Diet Quality Score (DQS) and subsequent classification of individuals into groups with overall *healthy, average* or *unhealthy* dietary habits. However, due to an error in uploading the survey to the online platform that resulted in the omission of one of the questions required for DQS calculation, typical fruit and vegetable (F&V) intake, which is an important predictor of general well-being and the risk of various diseases [7], was used as an indicator of diet quality. We assessed F&V intake both as a numeric outcome (portions per day; 1 portion = approx. 80 g) with higher intakes indicating better diet quality, and as a categorical variable (with three levels *less than 3 portions per day, 3 or 4 portions per day*, and *5 or more portions per day*) with meeting the ‘5-a-day’ target indicating sufficient F&V consumption [3]. We also asked if participants followed any meat-avoiding diet, and if so, what type (i.e. vegan, vegetarian, pescatarian or flexitarian), as reduced meat consumption has also been linked to better well-being and lower risk of certain diseases in higher income countries [11, 12]. Data was also collected on typical alcohol consumption (units per typical week, with four categories *rarely/ never drink alcohol*, *less than 14 units*, *between 14 & 21 units*, and *more than 21 units per week*, as defined by the SFFFQ), with 14 or more units per week considered to pose increasing risk to health as the closest approximation to the NHS’s definition of ‘increasing risk drinking’ being more than 14 units on a typical week [89].

#### Physical activity

Physical activity level was measured using the International Physical Activity Questionnaire (IPAQ) short format [90], which forms a part of the SFFFQ. Physical activity levels (*low, moderate*, or *high*) were assigned to participants based on the frequency, intensity and amount of exercise they had done in the previous week. For this, an estimate of their typical energy expenditure, as MET (Metabolic Equivalent of Task) Minutes per week, was calculated from self-reported amounts of exercise of different intensity. Total MET Minutes per week were calculated as the sum of MET Minutes for each exercise type (i.e. *light*, *moderate* or *vigorous*) undertaken by the participant in the previous week, obtained using the formula: *duration(mins) x frequency(days per week) x MET value (light exercise = 3.3, moderate exercise = 4, vigorous exercise = 8)*. Physical activity levels were assigned as follows: *high* if (a) vigorous activity on at least 3 days and achieving a total physical activity of at least 1500 MET Minutes per week OR (b) 7 or more days of any combination of light, moderate or vigorous activities achieving a total physical activity of at least 3000 MET Minutes per week; *moderate* if (a) 3 or more days of vigorous activity of at least 20 min per day OR (b) 5 or more days of moderate activity and/or light activity of at least 30 min per day OR (c) 5 or more days of any combination of light, moderate or vigorous activities achieving a total physical activity of at least 600 MET Minutes per week; *low* if not moderate or high. We used low physical activity level as an indicator of increased risk to physical [13, 14] and mental health [31].

#### Perceived effects of the pandemic

Participants were asked what kind of effect they felt the covid-19 pandemic had on their physical health, mental health, access to healthy food, and diet quality (individual questions with options *very negative, somewhat negative, neutral, somewhat positive* and *very positive*).

### Gardening related variables

Information about participants’ gardening habits used as independent variables included the number of hours spent gardening in a typical week (collected as a numerical, but for our analyses we used the categories 1‒5 h, 6‒10 h, 11 h or more, and 0 h for those who did not regularly garden), how much food they produced (on a self-rated scale of five, with 1 referring to a very small amount, 5 indicating virtual self-sufficiency in F&V, and 0 assigned to those who did not grow food), and whether they had an allotment.

### Demographic information

Socio-demographic information collected in the survey include gender, age, highest level of education, household income, household composition, caring responsibilities, Index of Multiple Deprivation (IMD) quintile (derived from participants’ postcodes), whether the participant had any long-term health conditions (assessed with a single *yes/no* type question), BMI (from reported height and weight, focusing on obesity as a risk factor), smoking (current and ex-smokers considered to be at increased risk, those who never smoked more than 100 cigarettes at low risk).

### Analyses

A series of hierarchical regression models were used to test the effects of gardening related variables on our chosen health and well-being measures adjusting for relevant socio-demographic factors, which were selected based on previous research and inspection of our data. Control variables used in the study include gender, age, highest level of education, household income, household composition, caring responsibilities, IMD quintile, whether the participant had any long-term health conditions (*yes/no*), BMI (focusing on obesity as a risk factor), smoking (current and ex-smokers considered to be at increased risk, those who never smoked more than 100 cigarettes at low risk), alcohol consumption (14 or more units per typical week considered ‘increasing risk drinking’), F&V intake (*less than 3 portions per day, 3 or 4 portions per day*, and *5 or more portions per day*), whether any meat-avoiding diet was followed and if so what type (vegan, vegetarian, pescatarian or flexitarian), and physical activity level (IPAQ category). Due to limitations imposed by sample size and the specific characteristics of the study population, the number of levels of certain factors (e.g. age, education level) were reduced. Weekly gardening time and level of food production were treated as categorical rather than numeric variables to allow comparisons with non-gardeners (who were assigned a value of zero for these variables) without violating model assumptions.

To test the effects of gardening related variables on continuous health and well-being outcomes (i.e. WEMWBs score, PHQ score, F&V intake), multiple linear regression models were used. For categorical outcomes (i.e. SRH, WEMWBS level, obesity, IPAQ level, 5-a-day F&V consumption, weekly outdoor time, health-related effects of the pandemic), multiple binary logistic regression models were used. To control for the potential confounding effects of socio-demographic and lifestyle factors, analyses were carried out in a hierarchical way. In the first step of each regression, a model adjusted for gender and age was fitted (Model 1). In step two, other key socio-demographic predictors were added to Model 1 and their significance in predicting the outcome was assessed. If any of these predictors had an associated *p* value of 0.1 or above, a new model was fitted with the predictor with the highest *p* value removed, the fit of the two models were compared using their Bayesian Information Criteria (BIC), and the model with the lower BIC was selected. If this model still contained predictors with effects with *p* ≥ 0.1, the process was repeated until further removal of predictors did not lead to an improvement in model fit (Model 2). In the third step of the regression, key risk factors to health were added to Model 2 and the above-described method was used to find the best fit model (Model 3). In the final step, variables related to gardening were introduced to Model 3 and the most parsimonious model was identified based on BIC (Model 4). Regression parameters (R^2^, B coefficients, standard error (SE), *p* values and, for logistic regression, χ^2^, odds ratios (OR) and 95% confidence intervals (CI)) were reported for the best fit models. We assessed parametric assumptions of no multicollinearity (based on Generalised Variance Inflation Factors (GVIF), using a threshold of 3) and, for linear regression, linearity (using scatter plots and residual diagnostics), and checked for the presence of potential outliers (based on standardised residual distribution) and influential cases (based on Cook’s distance and leverage plots) in each regression. To assess their generalisability, normality of standardised residual distribution and homogeneity of residual variance were also evaluated for linear regression models. Analyses were carried out in R (version 4.0.3).

## Results

### Participants

The study population (*N* = 280) comprised almost entirely (97.5%) white, predominantly female (74.6%) adults (Supplementary Table S1, Additional File 1). Nearly three quarters (72.5%) of respondents identified as regular gardeners, representing a similar proportion of both genders. Nearly half of all respondents were aged 55 or over, around 30% between 35 and 54, and 20% under 35 years old. Gardening was most common among people over 55, around 60% of whom were regular gardeners. The majority of respondents (78.2%) had received higher education. Participants were living in neighbourhoods representing all five English IMD quintiles but were predominantly from quintiles 3 to 5. The distribution of participants across IMD quintiles and household income categories was fairly similar among gardeners and non-gardeners. All of those who regularly gardened had a home garden, and around a half of regular gardeners and 36% of all participants had an allotment. Participants spent varying amounts of time gardening and were engaged in different levels of food production (Supplementary Table S2, Additional File 1).

### Predictors of health outcomes

#### Self-rated health (SRH)

According to our best fit logistic regression model, SRH in the study population was positively associated with growing food, and negatively with having obesity or long-term health conditions (Table 1). Participants with obesity were 8 times (OR = 7.98, 95%CI = 2.49–27.77, *p* < 0.001), and those with long-term health conditions over 18 times (OR = 18.57, 95%CI = 5.52–79.10, *p* < 0.001) more likely to have ‘not good’ health compared to people without obesity and without long-term conditions, respectively. Participants who grew moderate to large amounts of food (i.e. food growing levels 3, 4 and 5) were around 90% less likely (OR = 0.14, 95%CI = 0.02–0.64, *p* < 0.05; OR = 0.04, 95%CI = 0.00–0.41, *p* < 0.05; and OR = 0.13, 95%CI = 0.01–0.83, *p* < 0.05, respectively) to report ‘not good’ health compared to participants who did not grow food.

**Table 1:** Odds Ratios (OR) for ‘not good health’ as compared to ‘good health’ ^a, b, c, d^

Variable (reference category)	B (SE)	OR (95% CI)	p value
*Constant*	-2.04 (0.63)	0.13 (0.03–0.41)	< 0.01
Gender (Female)			
Male	-0.46 (0.67)	0.63 (0.16–2.24)	0.49
Age (18–34)			
35–54	0.34 (0.73)	1.40 (0.33–6.00)	0.65
55+	-0.27 (0.83)	0.76 (0.15–3.96)	0.74
Obesity (Not obese)			
Obese	2.08 (0.61)	**7.98 (2.49–27.77)**	< 0.001
Long-term conditions (No)			
Yes	2.92 (0.67)	**18.57 (5.52–79.10)**	< 0.001
Physical activity level (Low)			
Moderate	-1.19 (0.69)	0.30 (0.07–1.08)	0.08
High	-0.70 (0.94)	0.50 (0.06–2.73)	0.46
Food growing level (No food grown)			
1 (very little F&V)	-1.08 (0.91)	0.34 (0.05–1.87)	0.23
2	-1.53 (0.91)	0.22 (0.03–1.17)	0.09
3	-1.99 (0.83)	**0.14 (0.02–0.64)**	< 0.05
4	-3.27 (1.37)	**0.04 (0.00–0.41)**	< 0.05
5 (nearly self-sufficient in F&V)	-2.07 (1.01)	**0.13 (0.01–0.83)**	< 0.05

^a^ ‘Not good health’ refers to responses of ‘poor’ or ‘fair’ on the SRH, ‘good health’ includes responses of ‘good’, ‘very good’ or ‘excellent’

^b^ Predictors and regression coefficients in the table are derived from the best fit model for the outcome based on the Bayesian Information Criterion (BIC). Other explanatory variables tested include neighbourhood deprivation, household income, household composition, caring responsibilities, higher education, smoking status, alcohol consumption, F&V intake, time spent gardening, and having allotment, but these were dropped in the process of improving model fit

^c^ Model R^2^ = 0.39 (Hosmer Lemeshow), 0.30 (Cox and Snell), 0.50 (Nagelkerke); χ^2^ [12] = 66.45

^d^ Figures in bold are statistically significant at the 5% level (*p* < 0.05)

#### Mental well-being (WEMWBS)

Mental well-being among survey respondents was positively associated with physical activity level, gardening, and having an allotment, and negatively with neighbourhood deprivation and obesity. On average, those who spent 11 or more hours gardening in a typical week scored 4.57 points ± 1.92 (S.E.) higher on the WEMWBS than those who did not regularly garden (Table 2). In addition, participants who had an allotment were 67% less likely (OR = 0.33, 95%CI = 0.10–0.94, *p* < 0.05) to have low mental well-being than those without an allotment, while participants with obesity were 4.5 times more likely (OR = 4.46, 95%CI = 1.64–12.65, *p* < 0.01) to have low mental well-being compared to those without obesity (Table 3). The odds of having high mental well-being were not affected by variables related to gardening, but were positively associated with being male (OR = 10.57, 95%CI = 2.98–45.32, *p* < 0.001) and with having moderate or high, compared to low, physical activity levels (OR = 5.33, 95%CI = 1.06–31.66, *p* < 0.05; and OR = 5.55, 95%CI = 1.13–32.26, *p* < 0.05, respectively) (Supplementary Table S3, Additional File 1). Participants’ perception of the effect of the covid-19 pandemic on their mental health was negatively associated with having long-term health conditions, and positively with being aged 55 or over and with spending larger amounts of time gardening (Table 4). Those who spent at least 11 h gardening in a typical week were 78% less likely (OR = 0.22, 95%CI = 0.07–0.64, *p* < 0.01) than non-gardeners to report that the pandemic had a negative effect on their mental health.

**Table 2:** Hierarchical linear regression analysis of predictors of mental well-being (WEMWBS score) ^a, b, c, d^

Predictor variables (reference)	Model 1		Model 2		Model 3		Model 4	
	B (SE)	p	B (SE)	p	B (SE)	p	B (SE)	p
*Constant*	45.50 (1.35)	< 0.001	41.08 (2.91)	< 0.001	40.87 (3.08)	< 0.001	41.52 (3.16)	< 0.001
Gender (Female)								
Male	2.51 (1.38)	0.07	2.34 (1.36)	0.09	2.20 (1.35)	0.10	1.66 (1.34)	0.22
Age (18–34)								
35–54	1.79 (1.69)	0.29	1.80 (1.73)	0.30	1.93 (1.81)	0.29	1.09 (1.81)	0.55
55+	**5.96** (1.59)	< 0.001	**5.56** (1.77)	< 0.01	**5.20** (1.90)	< 0.01	3.25 (2.05)	0.11
IMD quintile (First)								
Second			**5.78** (2.76)	< 0.05	**6.13** (2.76)	< 0.05	**6.14** (2.72)	< 0.05
Third			1.14 (2.57)	0.66	1.84 (2.56)	0.47	1.69 (2.52)	0.50
Fourth			2.41 (2.59)	0.35	2.80 (2.57)	0.28	3.27 (2.54)	0.20
Fifth			2.04 (2.55)	0.42	2.09 (2.52)	0.41	2.59 (2.49)	0.30
Household (Alone)								
With partner			**3.91** (1.74)	< 0.05	**3.81** (1.70)	< 0.05	2.95 (1.71)	0.09
With family			0.55 (2.05)	0.79	0.80 (2.01)	0.69	0.46 (2.00)	0.82
Shared accommodation			4.81 (4.20)	0.25	4.95 (4.20)	0.24	4.37 (4.17)	0.30
Obesity (Not obese)								
Obese					-2.98 (1.61)	0.08	-2.45 (1.71)	0.15
Smoking (Non-smoker)								
Current- or ex-smoker					2.26 (1.30)	0.08	2.29 (1.30)	0.08
Daily F&V intake (5 + portions)								
1 or 2 portions					-5.01 (2.57)	0.05	-4.85 (2.55)	0.06
3 or 4 portions					0.29 (1.56)	0.85	-0.50 (1.57)	0.75
Weekly gardening time (0 h)								
1–5 h							-0.12 (1.63)	0.94
6–10 h							-0.28 (1.90)	0.89
11 + hours							**4.57** (1.92)	< 0.05
*R* ^*2*^	0.11		0.18		0.23		0.27	
*Adjusted R* ^*2*^	0.09		0.13		0.17		0.20	

^a^ Higher WEMWBS scores indicate better mental well-being

^b^ Model 1: adjusted for age and gender; Model 2: adjusted for sociodemographic variables; Model 3: adjusted for sociodemographic variables and health risk and relevant lifestyle factors; Model 4: adjusted for sociodemographic variables, health risk and relevant lifestyle factors and variables related to gardening

^c^ Predictors and coefficients in the table are derived from the best fit models for the outcome based on the Bayesian Information Criterion (BIC) at each stage of the regression. Other explanatory variables tested include household income, having higher education, drinking category, having long-term conditions, physical activity level, food growing, and having an allotment, but these were dropped in the process of improving model fit

^d^ Figures in bold are statistically significant at the 5% level (*p* < 0.05)

**Table 3:** Odds Ratios (OR) for low well-being (WEMWBS < 43) as compared to moderate well-being (WEMWBS 43–59) a, b, c

Variable (reference category)	B (SE)	OR (95% CI)	p value
*Constant*	-0.04 (0.72)	0.96 (0.23–4.03)	0.96
Gender (Female)			
Male	0.22 (0.49)	1.25 (0.47–3.18)	0.65
Age (18–34)			
35–54	-0.19 (0.51)	0.83 (0.30–2.24)	0.70
55+	-1.00 (0.58)	0.37 (0.11–1.12)	0.08
IMD quintile (First)			
Second	-1.51 (0.94)	0.22 (0.03–1.33)	0.11
Third	-0.82 (0.75)	0.44 (0.10–1.98)	0.28
Fourth	-0.48 (0.78)	0.62 (0.13–2.94)	0.54
Fifth	-1.15 (0.77)	0.32 (0.07–1.46)	0.14
Obesity (Not obese)			
Obese	1.50 (0.52)	**4.46 (1.63–12.65)**	< 0.01
Allotment (No)			
Yes	-1.12 (0.57)	**0.33 (0.10–0.94)**	< 0.05

^a^ Predictors and regression coefficients in the table are derived from the best fit model for the outcome based on the Bayesian Information Criterion (BIC). Other explanatory variables tested include household income, household composition, higher education, caring responsibilities, smoking status, alcohol consumption, long-term health conditions, F&V intake, physical activity level, time spent gardening, and growing food, but these were dropped in the process of improving model fit

^b^ Model R^2^ = 0.16 (Hosmer Lemeshow), 0.15 (Cox and Snell), 0.23 (Nagelkerke); χ^2^ (9) = 27.47

^c^ Figures in bold are statistically significant at the 5% level (*p* < 0.05)

**Table 4:** Odds Ratios (OR) for negative as compared to better effect of the pandemic on well-being ^a, b, c, d^

Variable (reference category)	B (SE)	OR (95% CI)	p value
*Constant*	-1.22 (0.44)	3.40 (1.50–8.41)	< 0.01
Gender (Female)			
Male	-0.48 (0.39)	0.62 (0.28–1.33)	0.23
Age (18–34)			
35–54	-0.75 (0.48)	0.47 (0.18–1.20)	0.12
55+	-1.74 (0.51)	**0.18 (0.06–0.47)**	< 0.001
Obesity (Not obese)			
Obese	0.77 (0.49)	2.15 (0.84–5.75)	0.11
Long-term conditions (No)			
Yes	0.79 (0.37)	**2.20 (1.09–4.63)**	< 0.05
Weekly gardening time (0 h)			
1–5 h	-0.53 (0.46)	0.59 (0.24–1.46)	0.25
6–10 h	-0.92 (0.53)	0.40 (0.14–1.11)	0.08
11 + hours	-1.53 (0.57)	**0.22 (0.07–0.64)**	< 0.01

^a^ ‘Negative’ self-reported effect of the covid-19 pandemic refers to responses of ‘very negative’ or ‘somewhat negative’, while ‘better’ includes responses of ‘neutral’, ‘somewhat positive’ or ‘very positive’

^b^ Predictors and regression coefficients in the table are derived from the best fit model for the outcome based on the Bayesian Information Criterion (BIC). Other explanatory variables tested include neighbourhood deprivation, household income, household composition, caring responsibilities, higher education, alcohol consumption, smoking status, physical activity level, F&V intake, food growing, and having an allotment, but these were dropped in the process of improving model fit

^c^ Model R^2^ = 0.18 (Hosmer Lemeshow), 0.22 (Cox and Snell), 0.30 (Nagelkerke); χ^2^ (8) = 46.94

^d^ Figures in bold are statistically significant at the 5% level (*p* < 0.05)

### Fruit and vegetable (F&V) intake, meat-avoidance, and diet-related effects of the pandemic

Typical daily F&V intake (number of 80 g portions) was positively associated with age, certain meat-avoiding diets, and growing food, and negatively with increasing-risk alcohol consumption (Tables 5 and 6). Compared to participants under 35, those aged 35–54 consumed on average 1.02 portions ± 0.41 (S.E.) more F&V daily, and were 3.6 times more likely (OR = 3.64, 95%CI = 1.30–10.74, *p* < 0.05) to meet the ‘5-a-day’ target (400 g), while those aged 55 or over consumed 1.16 portions ± 0.46 (S.E.) more F&V daily, and were 10.2 times more likely (OR = 10.22, 95%CI = 2.83–41.21, *p* < 0.001) to eat at least five portions of F&V on a typical day, genders showing no difference. Those following a flexitarian or pescatarian diet consumed 0.88 portions ± 0.34 (S.E.) and 1.87 portions ± 0.67 (S.E.), respectively, more F&V compared to regular meat-eaters, and flexitarians were also 2.9 times more likely (OR = 2.90, 95%CI = 1.10–8.34, *p* < 0.05) to meet the 5-a-day target than regular meat-eaters. Participants growing moderate to large amounts of food (i.e. food growing levels 3 and 4) consumed 1.21 portions ± 0.58 (S.E.) and 1.68 portions ± 0.69 (S.E.), respectively, more F&V daily than those who did not grow food, and those growing moderate amounts of food (i.e. food growing level 3) had 7.5 times higher odds of meeting the ‘5-a-day’ target compared to non-growers (OR = 7.47, 95%CI = 1.49–43.52, *p* < 0.05). The odds of following a meat-avoiding diet were not affected by variables related to gardening, but were positively associated with having higher education (OR = 3.61, 95%CI = 1.58–8.88, *p* < 0.01), and negatively with being male (OR = 0.43, 95%CI = 0.20–0.87, *p* < 0.05), and with living with a partner, compared to living alone (OR = 0.34, 95%CI = 0.13–0.82, *p* < 0.05) (Supplementary Table S4, Additional File 1).

**Table 5:** Hierarchical linear regression analysis of predictors of F&V intake adjusted for demographic and lifestyle factors ^a, b, c^

Predictor variables (reference)	Model 1		Model 2		Model 3		Model 4	
	B (SE)	p	B (SE)	p	B (SE)	p	B (SE)	p
*Constant*	4.54 (0.32)	< 0.001	4.54 (0.32)	< 0.001	4.08 (0.35)	< 0.001	3.99 (0.41)	< 0.001
Gender (Female)								
Male	-0.10 (0.33)	0.77	-0.10 (0.33)	0.77	0.27 (0.33)	0.41	0.15 (0.33)	0.65
Age (18–34)								
35–54	**1.16** (0.40)	< 0.01	**1.16** (0.40)	< 0.01	**1.24** (0.39)	< 0.05	**1.02** (0.41)	< 0.05
55+	**1.60** (0.38)	< 0.001	**1.60** (0.38)	< 0.001	**1.73** (0.37)	< 0.001	**1.16** (0.46)	< 0.05
Alcohol consumption (Low risk)								
Increasing risk					**-0.84** (0.35)	< 0.05	**-0.85** (0.36)	< 0.05
Diet (Meat-eater)								
Flexitarian					**0.90** (0.33)	< 0.01	**0.88** (0.34)	< 0.05
Pescatarian					**1.82** (0.67)	< 0.01	**1.87** (0.67)	< 0.01
Vegetarian					0.62 (0.44)	0.16	0.63 (0.45)	0.16
Vegan					1.00 (0.64)	0.12	0.98 (0.65)	0.13
Weekly gardening time (0 h)								
1–5 h							-0.94 (0.51)	0.06
6–10 h							-0.51 (0.60)	0.40
11 + hours							-0.44 (0.62)	0.47
Food growing level (No food grown)								
1 (very little F&V)							0.61 (0.56)	0.28
2							1.22 (0.62)	0.05
3							**1.21** (0.58)	< 0.05
4							**1.68** (0.69)	< 0.05
5 (nearly self-sufficient in F&V)							1.28 (0.67)	0.06
*R* ^*2*^	0.09		0.09		0.19		0.24	
*Adjusted R* ^*2*^	0.07		0.07		0.15		0.17	

^a^ Model 1: adjusted for age and gender; Model 2: adjusted for sociodemographic variables; Model 3: adjusted for sociodemographic variables and health risk and relevant lifestyle factors; Model 4: adjusted for sociodemographic variables, health risk and relevant lifestyle factors and variables related to gardening

^b^ Predictors and regression coefficients in the table are derived from the best fit model for the outcome based on the Bayesian Information Criterion (BIC). Other explanatory variables tested include neighbourhood deprivation, household income, household composition, caring responsibilities, having higher education, smoking status, physical activity level, long-term health conditions, and having an allotment, but these were dropped in the process of improving model fit

^c^ Figures in bold are statistically significant at the 5% level (*p* < 0.05)

**Table 6:** Odds Ratios (OR) for eating at least 5 F&V portions daily compared to fewer portions ^a, b, c^

Variable (reference category)	B (SE)	OR (95% CI)	p value
*Constant*	-1.95 (0.84)	0.14 (0.03–0.70)	< 0.05
Gender (Female)			
Male	-0.49 (0.44)	0.61 (0.26–1.47)	0.27
Age (18–34)			
35–54	1.29 (0.54)	**3.64 (1.30–10.74)**	< 0.05
55+	2.32 (0.68)	**10.22 (2.83–41.21)**	< 0.001
Household income (£20,000–29,999)			
Under £10,000	2.39 (1.38)	10.93 (0.96–295.67)	0.08
£10,000–19,999	-0.04 (0.77)	0.97 (0.22–4.55)	0.96
£30,000–39,999	0.22 (0.68)	1.25 (0.33–4.89)	0.75
£40,000+	-0.20 (0.61)	0.82 (0.24–2.69)	0.74
Higher education (No)			
Yes	0.96 (0.54)	2.61 (0.92–7.77)	0.07
Diet (Regular meat-eater)			
Flexitarian	1.06 (0.51)	**2.90 (1.10–8.34)**	< 0.05
Pescatarian	16.99 (1079.12)	2.39e^7^ (0.00–NA)	0.99
Vegetarian	0.81 (0.62)	2.25 (0.70–8.05)	0.19
Vegan	1.38 (0.90)	3.98 (0.77–30.68)	0.13
Weekly gardening time (0 h)			
1–5 h	-0.58 (0.69)	0.56 (0.14–2.08)	0.40
6–10 h	-0.80 (0.91)	0.45 (0.07–2.55)	0.38
11 + hours	-1.74 (0.96)	0.18 (0.02–1.05)	0.07
Food growing level (No food grown)			
1 (very little F&V)	0.16 (0.70)	1.17 (0.29–4.71)	0.82
2	0.91 (0.83)	2.49 (0.50–13.54)	0.27
3	2.01 (0.85)	**7.47 (1.49–43.52)**	< 0.05
4	2.00 (1.04)	7.38 (1.05–65.33)	0.05
5 (nearly self-sufficient in F&V)	1.84 (0.97)	6.33 (1.00–45.98)	0.06

^a^ Predictors and regression coefficients in the table are derived from the best fit model for the outcome based on the Bayesian Information Criterion (BIC). Other explanatory variables tested include neighbourhood deprivation, household composition, caring responsibilities, alcohol consumption, smoking status, obesity, long-term health conditions, and having an allotment, but these were dropped in the process of improving model fit

^b^ Model R^2^ = 0.25 (Hosmer Lemeshow), 0.26 (Cox and Snell), 0.38 (Nagelkerke); χ^2^ (2) = 57.54

^c^ Figures in bold are statistically significant at the 5% level (*p* < 0.05)

Participants’ perception of the effect of the pandemic on their diet quality or access to healthy food was not affected by gardening related variables but was associated with a number of socio-demographic factors. Respondents aged 55 or over and those with a household income of £30,000–39,999 were 95% less likely (OR = 0.05, 95%CI = 0.00–0.43 and OR = 0.05, 95%CI = 0.00–0.67, *p* < 0.05) than respondents under 35 and those with a household income of £20,000–29,999, respectively, to report experiencing a negative effect of the pandemic on their access to healthy food (Supplementary Table S5, Additional File 1). Participants aged 55 or over were also 92% less likely (OR = 0.08, 95%CI = 0.02–0.29, *p* < 0.001) than under 35s to report that the pandemic had a negative effect on their diet quality, while participants with obesity were around 4 times (OR = 4.21, 95%CI = 1.52–11.82, *p* < 0.01), and those typically consuming 1 or 2 portions of F&V daily were 9 times (OR = 9.09, 95%CI = 0.08–44.74, *p* < 0.01) more likely to report a pandemic-related negative effect on their diet quality when compared to those without obesity and those with a typical daily F&V intake of 5 or more portions, respectively (Supplementary Table S6, Additional File 1).

### Physical health (PHQ score)

Physical well-being, measured as the frequency of somatic health complaints, was positively associated with age and education level, and negatively with living with a family, having long-term health conditions, and increasing risk alcohol consumption (Table 7). Participants aged 35‒54 scored 5.70 points ± 1.97 (S.E.) lower (i.e. had less frequent health complaints), those aged 55 or over scored 11.11 points ± 2.22 (S.E.) lower on the PHQ than under 35s. Respondents with higher education scored 3.89 points ± 1.65 (S.E.) lower on the PHQ than those without higher education, while those living with a family scored 4.83 points ± 2.24 (S.E.) higher (i.e. had more frequent health complaints) than participants living alone. Participants with long-term health conditions or increasing risk drinking scored 4.21 points ± 1.37 (S.E.) and 4.43 points ± 1.73 (S.E.) higher than those without long-term conditions and with low-risk alcohol consumption, respectively. Participants who were aged 55 or over, had higher education or had moderate physical activity levels were less likely to report the pandemic having had a negative effect on their physical health compared to participants under 35, without higher education, and with low physical activity, respectively (Supplementary Table S7, Additional File 1). Neither PHQ scores nor the odds of attributing a negative physical health effect to the pandemic was associated with gardening related variables.

**Table 7:** Hierarchical linear regression analysis of predictors of physical health (PHQ) score ^a, b, c, d^

Predictor variables (reference)	Model 1			Model 2		Model 3		Model 4		
	B (SE)	p	B (SE)		p	B (SE)	p		B (SE)	p
*Constant*	41.37 (1.59)	< 0.001	48.34 (3.60)		< 0.001	45.31 (3.46)	< 0.001		45.43 (3.58)	< 0.001
Gender (Female)										
Male	-2.36 (1.63)	0.15	-1.64 (1.57)		0.30	-2.85 (1.53)	0.06		-2.69 (1.53)	0.08
Age (18–34)										
35–54	-3.55 (1.99)	0.08	**-4.48** (2.1)		< 0.05	**-6.08** (1.92)	< 0.01		**-5.70** (1.97)	< 0.01
55+	**-10.61** (1.86)	< 0.001	**-10.85** (2.07)		< 0.001	**-12.19** (1.99)	< 0.001		**-11.11** (2.22)	< 0.001
IMD quintile (First)										
Second			**-6.89** (3.14)		< 0.05	-5.21 (3.00)	0.08		-5.13 (3.00)	0.09
Third			-2.11 (2.96)		0.48	-1.41 (2.81)	0.62		-1.10 (2.82)	0.70
Fourth			-4.74 (3.01)		0.12	-2.90 (2.86)	0.31		-2.83 (2.86)	0.32
Fifth			**-6.04** (2.93)		< 0.05	-5.11 (2.77)	0.07		-4.94 (2.79)	0.08
Higher education (No)										
Yes			**-4.33** (1.74)		< 0.05	**-4.05** (1.64)	< 0.05		**-3.89** (1.65)	< 0.05
Household (Alone)										
With partner			-0.29 (2.04)		0.89	-0.79 (1.95)	0.69		-0.56 (1.97)	0.77
With family			4.64 (2.36)		0.05	**4.62** (2.24)	< 0.05		**4.83** (2.24)	< 0.05
Shared accommodation			-5.57 (4.52)		0.22	-5.39 (4.29)	0.21		-6.42 (4.38)	0.14
Obesity (Not obese)										
Obese						**3.65** (1.84)	< 0.05		3.26 (1.89)	0.09
Long-term conditions (No)										
Yes						**4.42** (1.35)	< 0.01		**4.21** (1.37)	< 0.01
Alcohol consumption (Low risk)										
Increasing risk						**4.47** (1.70)	< 0.01		**4.43** (1.73)	< 0.05
Weekly gardening time (0 h)										
1–5 h									4.21 (2.23)	0.06
6–10 h									1.97 (2.63)	0.45
11 + hours									2.89 (2.58)	0.26
Food growing (No)										
Yes									-4.32 (2.34)	0.07
*R* ^*2*^	0.18		0.29			0.38			0.40	
*Adjusted R* ^*2*^	0.17		0.25			0.33			0.34	

^a^ Lower PHQ scores indicate fewer health complaints and thus better health

^b^ Model 1: adjusted for age and gender; Model 2: adjusted for sociodemographic variables; Model 3: adjusted for sociodemographic variables and known health risk and relevant lifestyle factors; Model 4: adjusted for sociodemographic variables, health risk and relevant lifestyle factors and variables related to gardening

^c^ Predictors and coefficients in the table are derived from the best fit models for the outcome based on the Bayesian Information Criterion (BIC) at each stage of the regression. Other explanatory variables tested include household income, caring responsibilities, smoking status, physical activity level, F&V intake, and having an allotment, but these were dropped in the process of improving model fit

^d^ Figures in bold are statistically significant at the 5% level (*p* < 0.05)

### Obesity

The odds of having obesity were associated with age, physical activity level, F&V intake, and following a flexitarian diet (Table 8). Participants aged 35–54 were nearly 5 times more likely to have obesity than participants under 35 (OR = 4.83, 95%CI = 1.19–23.47, *p* < 0.05). Those with a moderate physical activity level were 72% less likely to have obesity than those with low activity levels (OR = 0.28, 95%CI = 0.07–0.91, *p* < 0.05), and those typically eating 3 or 4 portions of F&V were 3.9 times more likely (OR = 3.94, 95%CI = 1.22–13.74, *p* < 0.05) to have obesity than participants eating 5 or more portions of F&V daily. Flexitarians were also 87% (OR = 0.13, 95%CI = 0.03–0.49, *p* < 0.05) less likely to have obesity than regular meat-eaters. No gardening related variable had a significant effect on the odds of having obesity.

**Table 8:** Odds Ratios (OR) for having obesity as compared to being in any other BMI category ^a, b, c^

Variable (reference category)	B (SE)	OR (95% CI)	p value
*Constant*	-1.99 (0.80)	0.14 (0.03–0.61)	0.01
Gender (Female)			
Male	-1.03 (0.64)	0.36 (0.09–1.16)	0.11
Age (18–34)			
35–54	1.57 (0.75)	**4.83 (1.19–23.47)**	< 0.05
55+	0.45 (0.84)	1.56 (0.31–8.66)	0.60
Physical activity (Low)			
Moderate	-1.28 (0.64)	**0.28 (0.07–0.91)**	< 0.05
High	-0.51 (0.79)	0.60 (0.11–2.67)	0.52
Daily F&V intake (5 + portions)			
1 or 2 portions	1.35 (0.83)	3.85 (0.72–20.27)	0.11
3 or 4 portions	1.37 (0.61)	**3.94 (1.22–13.74)**	< 0.05
Diet (Regular meat-eater)			
Flexitarian	-2.01 (0.73)	**0.13 (0.03–0.49)**	< 0.01
Pescatarian	-16.25 (1239.58)	0.00 (NA–1.96e^34^)	0.99
Vegetarian	-1.34 (0.89)	0.26 (0.03–1.25)	0.13
Vegan	-1.49 (1.21)	0.22 (0.01–1.76)	0.22
Weekly gardening time (0 h)			
1–5 h	1.17 (0.87)	3.23 (0.62–19.84)	0.18
6–10 h	1.97 (1.10)	7.17 (0.95–72.37)	0.07
11 + hours	-0.09 (1.25)	0.92 (0.08–11.17)	0.94
Food growing level (No food grown)			
1 (very little F&V)	-1.86 (1.07)	0.16 (0.01–1.10)	0.08
2	-0.43 (1.01)	0.65 (0.08–4.49)	0.67
3	-0.84 (1.03)	0.43 (0.05–3.12)	0.42
4	-0.54 (1.16)	0.59 (0.06–5.58)	0.64
5 (nearly self-sufficient in F&V)	-1.33 (1.24)	0.27 (0.02–2.71)	0.28

^a^ Predictors and regression coefficients in the table are derived from the best fit model for the outcome based on the Bayesian Information Criterion (BIC). Other explanatory variables tested include neighbourhood deprivation, household income, higher education, household composition, caring responsibilities, smoking status, alcohol consumption, and having an allotment, but these were dropped in the process of improving model fit

^b^ Model R^2^ = 0.26 (Hosmer Lemeshow), 0.20 (Cox and Snell), 0.35 (Nagelkerke); χ^2^ [19] = 42.07

^c^ Figures in bold are statistically significant at the 5% level (*p* < 0.05)

### Low physical activity

The odds of having a low physical activity level were not affected by variables related to gardening, but were associated with age, household income, and having caring responsibilities (Table 9). On average, participants aged 35‒54 were 3.6 times more likely (OR = 3.56, 95%CI = 1.33–10.22, *p* < 0.05) to have low physical activity than under 35s. Participants with caring responsibilities were 60% less likely (OR = 0.40, 95%CI = 0.18–0.85, *p* < 0.05) than those without caring responsibilities, while participants with a household income of £40,000 per annum or higher were 64% less likely (OR = 0.34, 95%CI = 0.13–0.96, *p* < 0.05) than those with a household income of £20,000–29,999 to have low physical activity.

**Table 9:** Odds Ratios (OR) for having low physical activity as compared to higher (moderate or high) ^a, b, c^

Variable (reference category)	B (SE)	OR (95% CI)	p value
*Constant*	-0.15 (0.63)	0.86 (0.24–2.95)	0.81
Gender (Female)			
Male	0.09 (0.37)	1.10 (0.52–2.28)	0.81
Age (18–34)			
35–54	1.27 (0.52)	**3.56 (1.33–10.22)**	< 0.05
55+	-0.01 (0.56)	0.99 (0.33–3.00)	0.98
Household income (£20,000–29,999)			
Under £10,000	0.22 (0.91)	1.25 (0.22–8.53)	0.81
£10,000–19,999	-0.86 (0.58)	0.42 (0.13–1.29)	0.13
£30,000–39,999	-0.58 (0.54)	0.56 (0.19–1.61)	0.29
£40,000+	-1.01 (0.50)	**0.36 (0.13–0.96)**	< 0.05
Caring responsibilities (No)			
Yes	-0.91 (0.39)	**0.40 (0.18–0.85)**	< 0.05
Diet (Regular meat-eater)			
Flexitarian	0.13 (0.38)	1.14 (0.54–2.44)	0.73
Pescatarian	-1.83 (1.13)	0.16 (0.01–1.05)	0.11
Vegetarian	0.25 (0.51)	1.29 (0.47–3.50)	0.62
Vegan	-0.94 (0.80)	0.39 (0.07–1.75)	0.24
Food growing level (No food grown)			
1 (very little F&V)	-0.05 (0.66)	0.95 (0.25–3.44)	0.94
2	0.38 (0.59)	1.46 (0.45–4.73)	0.52
3	0.45 (0.54)	1.56 (0.55–4.54)	0.41
4	0.93 (0.64)	2.53 (0.73–9.20)	0.15
5 (nearly self-sufficient in F&V)	0.99 (0.59)	2.68 (0.86–8.73)	0.09

^a^ Predictors and regression coefficients in the table are derived from the best fit model for the outcome based on the Bayesian Information Criterion (BIC). Other explanatory variables tested include neighbourhood deprivation, household composition, higher education, smoking status, alcohol consumption, long-term health conditions, obesity, F&V intake, amount of time spent gardening, and having an allotment, but these were dropped in the process of improving model fit

^b^ Model R^2^ = 0.11 (Hosmer Lemeshow), 0.14 (Cox and Snell), 0.19 (Nagelkerke); χ^2^ (17) = 28.64

^c^ Figures in bold are statistically significant at the 5% level (*p* < 0.05)

## Discussion

Gardening offers several potential health and well-being benefits, but our understanding of the particular role of allotments and home gardens, and the effects of the level of engagement in gardening and involvement with food production has thus far been limited. Here, we quantitatively assess the relationship between home and allotment gardening in the UK and a range of indicators and predictors of health and well-being to fill some of the knowledge gaps in the field. Specifically, we investigated the effects of the amount of time spent gardening, level of food production, and having an allotment. We provide evidence that, after accounting for several potential confounders, gardening related variables are associated with better self-rated health, higher mental well-being, and increased fruit and vegetable (F&V) consumption. Higher F&V intake was in turn also associated with better self-rated health and decreased odds of having obesity. Thus, gardening had a positive association with four different aspects of health and well-being, directly or indirectly, via increased F&V consumption. Our analyses have also revealed that different aspects of health and well-being are associated with different aspects of gardening, which suggests that a number of distinct mechanisms are involved in delivering benefits.

We found that survey respondents who had an allotment had lower odds of having low mental well-being than those without an allotment, regardless of how much time they spent gardening. The fact that this positive association was observed after accounting for a range of potential confounding variables suggests that having an allotment is likely a predictor of well-being itself, rather than simply an indicator of differences in socio-economic status that impacted on well-being, which may have been expected based on trends of decreasing allotment availability with increasing neighbourhood deprivation [75]. We also found well-being scores to be positively associated with at least 11 h of weekly gardening, but not with smaller amounts, which suggests that getting a mental well-being benefit, at least in the form measured by the WEMWBS, might require more serious engagement gardening. Our results indicate that spending larger amounts of time gardening could improve well-being, and having an allotment in particular could help protect against low well-being, but may not be sufficient for achieving high (compared to moderate) well-being, the odds of which were not affected by gardening related variables. This is in line with previous research using the WEMWBS that found the odds of high and low well-being to be determined by different factors, for example, alcohol intake and obesity being associated with low, but not high mental well-being, and F&V intake associated with high well-being [92, 93]. In addition, we found 11 or more hours of weekly gardening to be associated with lower odds of attributing a negative mental health effect to the pandemic. This is in agreement with a recent study that found that contact with green spaces helped people cope with the negative mental well-being impacts of the covid-19 lockdowns [94].

Growing moderate to large amounts of F&V was associated with both higher self-reported average F&V intake and increased odds of meeting the 5-a-day target. This is consistent with the results of another piece of recent research in our group, where we studied year-long F&V production, purchases and losses in 85 food-grower households in the UK, and found median daily per capita F&V intake to be 70% higher than the national average [79]. Other studies have also found an association between involvement with gardening and increased F&V intake, but the underlying mechanisms have so far been unclear and research focusing on allotments and domestic gardens in the UK has so far been scarce. The results of the present study suggest that, in this context, gardening contributes to increased F&V intake only if it involves the production of considerable amounts of F&V. This indicates that higher F&V intake is a response to increased availability of F&V through own production, and that engagement in gardening that involves the production of no or only smaller amounts of food may not trigger a dietary change. It is worth noting too that during the covid-19 lockdowns differences in access to F&V between people who grew their own and those who did not could have been more pronounced than under normal circumstances.

Nonetheless, we should not dismiss the idea that close exposure to a variety of F&V through own-growing could build familiarity and promote positive changes in diet, as some research suggests that this could be an effective mechanism for improving food behaviours, especially in children [61, 95, 96]. Although the underlying mechanisms are not fully understood, nature relatedness has also been linked to increased F&V intake [97], therefore engaging with natural processes through gardening may be an additional pathway through which food gardening can promote F&V consumption. Although we did not find evidence for a role of F&V gardening in alleviating the potential adverse effects of the pandemic on people’s access to healthy food or diet quality associated with temporary store closures and supply shortages [98], this may be due to the overall relatively small number of participants reporting negative experiences in these areas.

Growing moderate to large amounts of F&V was also associated with considerably lower odds of reporting ‘not good’ health, which suggests that a health benefit is mediated by increased F&V intake associated with access through own-growing. The importance of sufficient F&V consumption for the prevention of a range of non-communicable diseases is well established [3, 8]. Diets low in F&V are estimated to contribute to 18,000 premature deaths in the UK each year [19], so increasing F&V intake through own production could have important positive implications for public health. As the current cost of living crisis continues, increased availability of F&V through own-growing could particularly benefit people on lower incomes.

Although we did not find a direct association between the odds of having obesity and variables related to gardening, we did find evidence of an inverse relationship between obesity and F&V intake, in agreement with the literature [99, 100]. In addition, while there was no significant association between gardening and the odds of having low physical activity as measured in our study, previous research has established that many gardening tasks require moderate-intensity physical exercise [101]. Combined with a healthy diet, exercise can aid weight loss [102, 103], and evidence of an association between physical activity level and the odds of obesity was also present in our study. Thus, by promoting consumption of F&V and providing physical exercise, food gardening on allotments and in domestic gardens could contribute to the prevention of obesity and lower the risk of associated diseases. Moreover, we found a negative association between obesity and mental well-being, in accordance with previous research [92], suggesting an additional way in which gardening could improve well-being. The potential role of a healthy diet characterised by high consumption of F&V and moderate intake of animal products in improving mental well-being via several biological pathways, including the maintenance of a healthy body weight, has also been proposed in the literature [104].

Gardening is an outdoor activity by nature, which has been linked to less sedentary time, higher levels of physical activity, better self-rated health and decreased chronic disease risk [91, 105, 106]. Research on the effects of being outdoors and participating in physical activity in natural environments also point to benefits for psychological well-being [32, 40, 41, 107], some evidence of which was also present in our study. Therefore, as a form of green exercise, gardening could improve both physical and mental well-being, which may have been particularly important during the covid-19 lockdowns, which had a negative effect on many people’s physical activity levels [108] and mental well-being [28].

One somewhat unexpected finding from our study is that older participants had lower, rather than higher, as anticipated based on previous research, odds of reporting ‘not good’ health [109]. This might be related to the fact that in our sample there were notably more gardeners than non-gardeners among those aged 55 or over. However, the age effect persisted even after adjusting for the positive effect of gardening time, which suggests that there are likely some other factors associated with age that positively affect SRH that we did not control for in our analyses. To investigate this further, it would be valuable to repeat the study on a larger and more balanced sample.

### Strengths and limitations

Our study adds particular value to existing literature on the benefits of gardening by focusing on the role of allotments and home gardens in the UK, which has been relatively understudied compared to community gardens in the USA, and by examining the effects of the amount of time spent gardening, level of food production, and having an allotment, on a range of health-related outcomes. Nevertheless, because of our moderate sample size, some care should be taken when generalising findings beyond the study population, which is not fully representative of the UK general population. In addition, some categories of the variables studied were represented by relatively few people, preventing us from testing certain potentially significant associations. Further work is required to unravel the complex relationships between individual and societal determinants of health and well-being and how the role of gardening may vary with other factors. It is also important to remember that this research was conducted during the covid-19 pandemic while major restrictions were in place in the UK and the lives of people were majorly affected in different ways. While our study offers valuable insight into the links between gardening and health and well-being, these associations may be different under normal circumstances, the specifics of which need further exploration.

## Conclusions

Good health is an asset that has major impacts on both the individual and societal level. However, rates of mental and physical ill health and unhealthy behaviours that contribute to these are high and rising around the world [6]. Our study provides evidence that gardening on allotments and in domestic gardens in the UK could promote physical and mental well-being and help reduce the risk of a number of health conditions via various pathways and may have played a role in protecting against some of the negative impacts of the covid-19 pandemic. However, although gardening is a fairly popular activity in the UK, many people do not have access to a garden [110] and the growing demand for allotments is unmet by the dwindling current supply, especially in already deprived neighbourhoods [72, 111]. Improving access to growing space and promoting regular gardening could therefore provide a range of benefits to public health. There is also a need for more research to determine causal relationships and better understand how the effects of gardening may vary with socio-economic factors to guide policy-makers in devising strategies that help maximise its health and well-being benefits.

### Electronic supplementary material

Below is the link to the electronic supplementary material.

Supplementary Material 1

Supplementary Material 2

## Data Availability

The datasets generated and analysed in this study are not publicly available due to participant consent reasons. For more information, contact Dr Boglarka Zilla Gulyas (email: b.z.gulyas@sheffield.ac.uk).
