# Supplementary material for: Quantifying the relationship between gardening and health and well-being in the UK: a survey during the covid-19 pandemic
Source: BMC Public Health. 2024 Mar 14;24:810. doi: 10.1186/s12889-024-18249-8 (PMC10941614; doi:10.1186/s12889-024-18249-8)
Supplement: Supplementary file 1 — Supplementary Material 1 [file 12889_2024_18249_MOESM1_ESM.docx]

**Additional File 2**

**QUESTIONNAIRE USED TO COLLECT DATA FOR THE STUDY**

**Quantifying the relationship between gardening and health and well-being in the UK: a survey during the covid-19 pandemic**

Boglarka Z. Gulyas ^1,2^, Samantha J. Caton ^2^ and Jill L. Edmondson *^1^

^1^ Plants, Photosynthesis and Soil, School of Biosciences, University of Sheffield, Sheffield S10 2TN, UK

^2^ Sheffield Centre for Health and Related Research (SCHARR), School of Medicine and Population Health, University of Sheffield, Sheffield S10 2TN, UK

* Corresponding author. Email: j.edmondson@sheffield.ac.uk

**CONTENT**

**Section 1 Demographics**

**Section 2 Diet**

**Section 3 Access to green and growing space**

**Section 4 Gardening**

**Section 5 Wellbeing**

**Section 6 Impact of Covid-19**

**Section 1 - Demographics**

*Please provide some general information about yourself.*

How would you describe your gender?

Female / Male / Other

What is your age?

18-24 / 25-34 / 35-44 / 45-54 / 55-64 / 65+

What is your ethnic background?

White / Mixed / Asian or Asian British / Black or Black British / Arab / Other / Prefer not to say

What is your highest level of education?

No qualifications / GCSEs or equivalent / A levels or equivalent / Undergraduate degree / Postgraduate degree

What is your postcode?

___

Who do you live with?

Living alone / With partner / With family / In shared accommodation

How many people are there in your household (including yourself)?

___

What is your housing status?

Home-owner (outright) / Home-owner (with mortgage) / Renting / Living with family or at a friend’s house / Other

What is your annual household income?

Under 10,000 / 10,000-19,999 / 20,000-29,999 / 30-39,999 / 40,000 or over

What is your employment status?

Student / Full-time employed / Part-time employed / Full-time self-employed / Part-time self-employed / Temporarily unemployed / Long-term unemployed / Retired / Unable to work

Do you have caring responsibilities (including dependent children and elderly or disabled relatives)?

Yes / No

What is your height in centimetres?

___ cm

What is your weight in kilograms?

___ kg

Regarding your meat and dairy consumption, are you…?

Vegan / Vegetarian / Pescetarian (i.e. eat fish but not meat) / Flexitarian (i.e. only eat meat occasionally) / None of these

**Section 2 - Diet**

***(From the Short Form Food Frequency Questionnaire (SFFFQ) by Cleghorn et al.)***

*The following questions ask about some foods & drinks you might have* ***during a ‘typical’ week, over the past month or so****. Do not be concerned if some things you eat or drink are not mentioned.*

2. Please tick how often you eat at least ONE portion of the following foods & drinks: (a portion includes: a handful of grapes, an orange, a serving of carrots, a side salad, a slice of bread, a glass of pop). (Please only put one tick, but answer EVERY line)

*Answer options: Rarely or never / Less than 1 a Week / Once a Week / 2-3 times a Week / 4-6 times a Week / 1-2 times a Day / 3-4 times a Day / 5+ a Day*

Fruit (tinned / fresh)

Fruit juice (not cordial or squash)

Salad (not garnish added to sandwiches)

Vegetables (tinned / frozen / fresh but not potatoes)

Chips / fried potatoes

Beans or pulses like baked beans, chick peas, dahl

Fibre-rich breakfast cereal, like Weetabix, Fruit ‘n Fibre, Porridge, Muesli

Wholemeal bread or chapattis

Cheese / yoghurt

Crisps / savoury snacks

Sweet biscuits, cakes, chocolate, sweets

Ice cream / cream

Non-alcoholic fizzy drinks/pop (not sugar free or diet)

*Answer options: Rarely or never / Less than 1 a Week / Once a Week / 2-3 times a Week / 4-6 times a Week / 7+ times a week*

Whole meats:

Beef, Lamb, Pork, Ham - steaks, roasts, joints, mince or chops

Chicken or Turkey – steaks, roasts, joints, mince or portions (not in batter or breadcrumbs)

Processed meats/ meat products:

Sausages, bacon, corned beef, meat pies/pasties, burgers

Chicken/turkey nuggets/twizzlers, turkey burgers, chicken pies, or in batter or breadcrumbs

Fish:

White fish in batter or breadcrumbs – like ‘fish ‘n chips’

White fish not in batter or breadcrumbs

Oily fish – like herrings, sardines, salmon, trout, mackerel, fresh tuna (not tinned tuna)

3. On average, how many portions of FRUIT do you eat a day? (examples include a handful of grapes, an orange, a glass of fruit juice, a handful of dried fruits).

___

4. On average, how many portions of VEGETABLES do you eat a day? (examples include: 3 heaped tablespoons of carrots, a side salad, 2 spears of broccoli).

___

5. What milk do you usually use or drink, such as in hot & cold drinks or on cereal? (including tea, coffee, hot milk, milk shakes, or on cereal)

*Whole/full-fat milk / Semi-skimmed milk / Skimmed milk / Rarely/never use milk /*

*Other (please write its name)*

6. On average, how much alcohol do you drink over a complete seven day week? (One unit is a standard glass of wine, half a pint of beer or lager, a single measure of spirits, a measure of sherry)

*I rarely/never drink alcohol / Less than 14 units / Between 14 & 21 units / More than 21 units*

7. Last week (or a week before that if you have been poorly, self-isolating, or on holiday), how much exercise did you do, if any?

(a) I didn’t do any exercise

(b) I did some LIGHT exercise

This was fairly easy and I didn’t get out of breath (e.g. gentle walking, playing bowls or snooker, light DIY/housework).

I did this ________ times during the week.

Each time I did this lasted about ___________ minutes

AND / OR

(c) I did some MODERATE exercise

This made my breathing a little harder or made me sweat. (e.g. fast walking, swimming, golf, heavy housework).

I did this ________ times during the week.

Each time I did this lasted about ___________ minutes

AND / OR

(d) I did some VIGOROUS exercise

This made my breathing hard & made me sweat. (e.g. running, squash, hard swimming, aerobics).

I did this ________ times during the week.

Each time I did this lasted about ___________ minutes

8. Regarding smoking, are you?

A current smoker / An ex-smoker / I have never smoked more than 100 cigarettes

**Section 3 -** **Access to green and growing space**

*The following questions will ask about your access to and use of different types of green spaces around where you live.*

1. Do you have access to a garden?

Yes, I have access to a private garden / Yes, I have access to a shared garden / No

2. Do you have a balcony or terrace?

Yes / No

3. Are there any green areas such as parks or forests within easy walking distance from where you live?

Yes, one / Yes, more than one / No

4. Are there any allotment sites near where you live?

Yes / No / I don’t know

5. Are there any community gardens near where you live?

Yes / No / I don’t know

6. Do you have an allotment?

Yes / No

8. How much time did you spend outdoors in a typical week, over the past month or so?

Less than 3 hours / 3-6 hours / 7-10 hours / 11-14 hours / More than 14 hours

**Section 4 - Gardening**

*The following questions are related to gardening.*

1. Do you regularly engage in gardening activities?

Yes / No

2. Does anyone in your household garden?

Yes / No

3. How many times did you do garden work in a typical week over the past month or so?

___ days per week

4. How much time did you spend gardening in a typical week over the past month or so?

___ hours per week

5. How long have you been gardening for?

___ years

6. What is/are your motivation(s) for gardening?

___ / Not applicable

7. Do you grow food (fruits, vegetables or herbs)?

Yes / No

8. Does anyone in your household grow food?

Yes / No

9. If you grow food, where?

In my garden / On an allotment / At a community garden / On my terrace or balcony / Other (please specify) / I don’t grow food

10. How long have you been growing food for?

___ years

11. How much food do you grow? Please rate on a scale of ten, with 1 being a very small amount (e.g. a couple of herbs in pots) and 5 implying near self-sufficiency in fruits and vegetables during most of the year.

1 / 2 / 3 / 4 / 5

12. What is/are your main motivation(s) for growing food?

___ / Not applicable

**Section 5 - Wellbeing**

*The following questions will ask about aspects of your health and wellbeing.*

**a) Overall health**

1. In general, how would you rate your health in the past year?

Excellent / Very good / Good / Fair / Poor

2. Do you have any chronic (long-term) health conditions?

Yes / No

**b) Physical wellbeing**

***(The Physical Health Questionnaire (PHQ) by Schat & Kelloway)***

*The following items focus on how you have been feeling physically during the past month.*

Answer options:

- *Qs 1-11: Not at all / Rarely / Once in a while / Some of the time / Fairly often / Often / All of the time*
- *Qs 12 and 13: 0 times / 1–2 time / 3 times / 4 times / 5 times / 6 times / 7+ times*
- *Q 14: 1 day / 2 days / 3 days / 4 days / 5 days / 6 days / 7+ days*

1. How often have you had difficulty getting to sleep at night?
2. How often have you woken up during the night?
3. How often have you had nightmares or disturbing dreams?
4. How often has your sleep been peaceful and undisturbed?
5. How often have you experienced headaches?
6. How often did you get a headache when there was a lot of pressure on you to get things done?
7. How often did you get a headache when you were frustrated because things were not going the way they should have or when you were annoyed at someone?
8. How often have you suffered from an upset stomach (indigestion)?
9. How often did you have to watch that you ate carefully to avoid stomach upsets?
10. How often did you feel nauseated (“sick to your stomach”)?
11. How often were you constipated or did you suffer from diarrhea?
12. How many times have you had minor colds (that made you feel uncomfortable but didn't keep you sick in bed or make you miss work)?
13. How many times have you had respiratory infections more severe than minor colds that “laid you low” (such as bronchitis, sinusitis, etc.)?
14. When you had a bad cold or flu, how long did it typically last?

Were you self-isolating some of the time in the past month?

**c) Mental wellbeing**

***(The Warwick-Edinburgh Mental Wellbeing Scale (WEMWBS))***

*Below are some statements about feelings and thoughts. Please select the answer that best describes your experience of each over the last 2 weeks.*

*Answer options: None of the Time / Rarely / Some of the Time / Often / All of the time*

1. I’ve been feeling optimistic about the future
2. I’ve been feeling useful
3. I’ve been feeling relaxed
4. I’ve been feeling interested in other people
5. I’ve had energy to spare
6. I’ve been dealing with problems well
7. I’ve been thinking clearly
8. I’ve been feeling good about myself
9. I’ve been feeling close to other people
10. I’ve been feeling confident
11. I’ve been able to make up my own mind about things
12. I’ve been feeling loved
13. I’ve been interested in new things
14. I’ve been feeling cheerful

Were you self-isolating some of the time in the past two weeks?

**Section 6 -** **Impact of Covid-19**

This section is about the impact of the Covid-19 pandemic on different areas of your life.

1. For each item below, please mark whether you feel like the pandemic has had a *very negative, somewhat negative, neutral, somewhat positive* or *very positive* effect.

Income

Career/work life

Amount of free time

Quality of free time

Relationship with family

Relationship with friends

Access to fresh and healthy food

Diet quality

Overall physical health

Overall mental health

Overall quality of life

2. Describe briefly how the pandemic has affected your life (optional):

___
