# Supplementary material for: Quantifying the relationship between gardening and health and well-being in the UK: a survey during the covid-19 pandemic
Source: BMC Public Health. 2024 Mar 14;24:810. doi: 10.1186/s12889-024-18249-8 (PMC10941614; doi:10.1186/s12889-024-18249-8)
Supplement: Supplementary file 2 — Supplementary Material 2 [file 12889_2024_18249_MOESM2_ESM.docx]

**Additional File 1**

**SUPPORTING INFORMATION**

**Quantifying the relationship between gardening and health and well-being in the UK: a survey during the covid-19 pandemic**

Boglarka Z. Gulyas ^1,2^, Samantha J. Caton ^2^ and Jill L. Edmondson *^1^

^1^ Plants, Photosynthesis and Soil, School of Biosciences, University of Sheffield, Sheffield S10 2TN, UK

^2^ Sheffield Centre for Health and Related Research (SCHARR), School of Medicine and Population Health, University of Sheffield, Sheffield S10 2TN, UK

* Corresponding author. Email: j.edmondson@sheffield.ac.uk

**CONTENT**

**Table S1 Socio-demographic characteristics of survey respondents (N=280), for gardeners and non-gardeners.**

**Table S2 Characteristics of survey respondents (N=280) related to gardening.**

**Table S3 Odds Ratios (OR) for high mental well-being (WEMWBS 60+) as compared to moderate mental well-being (WEMWBS 43–59), adjusted for demographic and lifestyle factors.**

**Table S4 Odds Ratios (OR) for following a meat-avoiding diet as compared to having a regular diet including meat, adjusted for demographic and lifestyle factors.**

**Table S5 Odds Ratios (OR) for negative (‘very negative’ or ‘somewhat negative’) as compared to neutral or positive (‘neutral’, ‘somewhat positive’ or ‘very positive’) self-reported effect of the covid-19 pandemic on participants’ access to healthy food, adjusted for demographic and lifestyle variables.**

**Table S6 Odds Ratios (OR) for negative (‘very negative’ or ‘somewhat negative’) as compared to neutral or positive (‘neutral’, ‘somewhat positive’ or ‘very positive’) self-reported effect of the covid-19 pandemic on participants’ diet quality, adjusted for demographic and lifestyle variables.**

**Table S7 Odds Ratios (OR) for negative (‘very negative’ or ‘somewhat negative’) as compared to neutral or positive (‘neutral’, ‘somewhat positive’ or ‘very positive’) self-reported effect of the covid-19 pandemic on physical health, adjusted for demographic and lifestyle factors.**

**Table S1** Socio-demographic characteristics of survey respondents (N=280), for gardeners and non-gardeners

|  | **Gardening** | | **Non-gardening** | | **TOTAL** | |
| --- | --- | --- | --- | --- | --- | --- |
|  | **n** | **%** | **n** | **%** | **n** | **%** |
| **TOTAL** | 203 | 72.5% | 71 | 25.4% | 280 | 100.0% |
| **Gender** |  |  |  |  |  |  |
| Male | 49 | 23.4% | 21 | 29.6% | 71 | 25.4% |
| Female | 154 | 73.7% | 50 | 70.4% | 209 | 74.6% |
| **Age** |  |  |  |  |  |  |
| 18–34 | 24 | 11.8% | 34 | 47.9% | 59 | 21.1% |
| 35–54 | 54 | 26.6% | 28 | 39.4% | 83 | 29.6% |
| 55+ | 125 | 61.6% | 9 | 12.7% | 138 | 49.3% |
| **Education** |  |  |  |  |  |  |
| A levels or lower | 44 | 21.7% | 15 | 21.1% | 61 | 21.8% |
| Postgraduate degree | 159 | 78.3% | 56 | 78.9% | 219 | 78.2% |
| **Household composition** |  |  |  |  |  |  |
| Alone | 33 | 16.3% | 7 | 9.9% | 41 | 14.6% |
| With partner | 122 | 60.1% | 31 | 43.7% | 156 | 55.7% |
| With family | 45 | 22.2% | 27 | 38.0% | 73 | 26.1% |
| In shared accommodation | 2 | 2.5% | 6 | 8.5% | 9 | 3.2% |
| **Caring responsibilities** | |  |  |  |  |  |
| Yes | 60 | 29.6% | 25 | 35.3% | 85 | 30.4% |
| No | 142 | 70.0% | 46 | 64.8% | 194 | 69.3% |
| **Household income** | |  |  |  |  |  |
| Under £10,000 | 7 | 3.5% | 3 | 4.2% | 10 | 3.6% |
| £10,000–19,999 | 29 | 14.3% | 3 | 4.2% | 32 | 11.4% |
| £20,000–29,999 | 47 | 23.2% | 11 | 15.5% | 61 | 21.8% |
| £30,000–39,999 | 34 | 16.3% | 11 | 15.5% | 47 | 16.8% |
| £40,000+ | 76 | 37.4% | 42 | 59.2% | 119 | 42.5% |
| **IMD quintile** |  |  |  |  |  |  |
| 1 | 11 | 5.4% | 4 | 5.6% | 16 | 5.7% |
| 2 | 22 | 10.8% | 8 | 11.3% | 30 | 10.7% |
| 3 | 46 | 22.7% | 12 | 16.9% | 59 | 21.1% |
| 4 | 36 | 17.7% | 7 | 9.9% | 45 | 16.1% |
| 5 | 47 | 23.2% | 22 | 31.0% | 70 | 25.0% |

**Table S2** Characteristics of survey respondents (N=280) related to gardening

|  | **n** | **%** |
| --- | --- | --- |
|  |  |  |
| **TOTAL** | 280 | 100.0% |
| **Weekly gardening time** |  |  |
| 0 hours | 72 | 25.7% |
| 1–5 hours | 75 | 26.8% |
| 6–10 hours | 56 | 20.0% |
| 11+ hours | 66 | 23.6% |
| **Food growing level** |  |  |
| No food grown | 65 | 23.2% |
| 1 (very little F&V) | 25 | 8.9% |
| 2 | 30 | 10.7% |
| 3 | 67 | 23.9% |
| 4 | 30 | 10.7% |
| 5 (nearly self-sufficient in F&V) | 54 | 19.3% |
| **Allotment** | |  |
| Yes | 102 | 36.4% |
| No | 172 | 63.6% |

**Table S3** Odds Ratios (OR) for high mental well-being (WEMWBS 60+) as compared to moderate mental well-being (WEMWBS 43–59), adjusted for demographic and lifestyle factors^a, b, c^

| **Variable (reference category)** | **B (SE)** | **OR (95% CI)** | ***p* value** |
| --- | --- | --- | --- |
| *Constant* | -5.01 (1.47) | 0.01 (0.00–0.08) | <0.001 |
| Gender (Female) |  |  |  |
| Male | 2.36 (0.68) | **10.57 (2.98–45.32)** | <0.001 |
| Age (18–34) |  |  |  |
| 35–54 | -0.12 (1.38) | 0.89 (0.07–23.16) | 0.93 |
| 55+ | 1.16 (1.17) | 3.20 (0.43–67.07) | 0.32 |
| Household income (£20,000–29,999) |  |  |  |
| Under £10,000 | -15.51 (1828.10) | 0.00 (NA–1.48e^+75^) | 0.99 |
| £10,000–19,999 | -1.35 (1.07) | 0.26 (0.02–1.83) | 0.21 |
| £30,000–39,999 | 0.11 (0.84) | 1.11 (0.20–5.88) | 0.90 |
| £40,000+ | -0.73 (0.84) | 0.48 (0.09–2.47) | 0.39 |
| Smoking (Non-smoker) |  |  |  |
| Current- or ex-smoker | 1.31 (0.69) | 3.71 (0.99–15.36) | 0.06 |
| IPAQ category (Low) |  |  |  |
| Moderate | 1.67 (0.85) | **5.33 (1.06–31.66)** | <0.05 |
| High | 1.71 (0.84) | **5.55 (1.13–32.26)** | <0.05 |
| ^a^ Predictors and regression coefficients in the table are derived from the best fit model for the outcome based on the Bayesian Information Criterion (BIC). Other explanatory variables tested include neighbourhood deprivation, household composition, caring responsibilities, higher education, obesity, alcohol consumption, long-term health conditions, F&V intake, time spent gardening, food growing level, and having an allotment, but these were dropped in the process of improving model fit.  ^b^ Model R^2^ = 0.31 (Hosmer Lemeshow), 0.19 (Cox and Snell), 0.38 (Nagelkerke); χ^2^ (10) = 30.84  ^C^ Figures in bold are statistically significant at the 5% level (p<0.05) | | | |

**Table S4** Odds Ratios (OR) for following a meat-avoiding diet as compared to having a regular diet including meat, adjusted for demographic and lifestyle factors^a, b, c^

| **Variable (reference category)** | **B (SE)** | **OR (95% CI)** | ***p* value** |
| --- | --- | --- | --- |
| *Constant* | -0.49 (0.65) | 0.62 (0.17–2.21) | 0.46 |
| Gender (Female) |  |  |  |
| Male | -0.85 (0.37) | **0.43 (0.20–0.87)** | <0.05 |
| Age (18-34) |  |  |  |
| 35-54 | -0.06 (0.46) | 1.06 (0.43–2.62) | 0.90 |
| 55+ | 0.47 (0.47) | 1.60 (0.64–4.12) | 0.32 |
| Higher education (No) |  |  |  |
| Yes | 1.28 (0.44) | **3.61 (1.58–8.88)** | <0.01 |
| Household (Alone) |  |  |  |
| With partner | -1.09 (0.46) | **0.34 (0.13–0.82)** | <0.05 |
| With family | -0.29 (0.54) | 0.75 (0.26–2.13) | 0.59 |
| Shared accommodation | 1.85 (1.29) | 6.33 (0.65–152.12) | 0.15 |
| ^a^ Predictors and regression coefficients in the table are derived from the best fit model for the outcome based on the Bayesian Information Criterion (BIC). Other explanatory variables tested include neighbourhood deprivation, household income, household composition, caring responsibilities, alcohol consumption, smoking status, obesity, long-term health conditions, time spent gardening, food-growing level, and having an allotment, but these were dropped in the process of improving model fit.  ^b^ Model R^2^ = 0.09 (Hosmer Lemeshow), 0.12 (Cox and Snell), 0.16 (Nagelkerke); χ^2^ (7) = 24.27  ^C^ Figures in bold are statistically significant at the 5% level (p<0.05) | | | |

**Table S5** Odds Ratios (OR) for negative (‘very negative’ or ‘somewhat negative’) as compared to neutral or positive (‘neutral’, ‘somewhat positive’ or ‘very positive’) self-reported effect of the covid-19 pandemic on participants’ access to healthy food, adjusted for demographic and lifestyle variables^a, b, c^

| **Variable (reference category)** | **B (SE)** | **OR (95% CI)** | ***p* value** |
| --- | --- | --- | --- |
| *Constant* | 0.64 (1.42) | 1.89 (0.11–31.78) | 0.65 |
| Gender (Female) |  |  |  |
| Male | -0.61 (0.78) | 0.54 (0.10–2.26) | 0.44 |
| Age (18–34) |  |  |  |
| 35–54 | -0.06 (0.70) | 0.95 (0.24–3.90) | 0.94 |
| 55+ | -3.03 (1.23) | **0.05 (0.00–0.43)** | <0.05 |
| IMD quintile (First) |  |  |  |
| Second | -1.18 (1.17) | 0.31 (0.03–3.16) | 0.31 |
| Third | -1.81 (1.23) | 0.16 (0.01–1.89) | 0.14 |
| Fourth | -169 (1.27) | 0.19 (0.01–2.21) | 0.18 |
| Fifth | -1.09 (1.17) | 0.34 (0.03–3.77) | 0.35 |
| Household income (£20,000–29,999) |  |  |  |
| Under £10,000 | -18.06 (1844.17) | 0.00 (NA–9.36e^37^) | 0.99 |
| £10,000–19,999 | -0.29 (1.13) | 0.75 (0.07–6.78) | 0.80 |
| £30,000–39,999 | -3.07 (1.55) | **0.05 (0.00–0.67)** | <0.05 |
| £40,000+ | -0.94 (0.99) | 0.39 (0.05–2.87) | 0.34 |
| Higher education (No) |  |  |  |
| Yes | -1.06 (0.96) | 0.35 (0.05–2.33) | 0.27 |
| Obesity (Not obese) |  |  |  |
| Obese | 1.16 (0.66) | 3.19 (0.85–11.82) | 0.08 |
| Daily F&V intake (5+ portions) |  |  |  |
| 1 or 2 portions | 0.69 (1.02) | 2.00 (0.22–14.05) | 0.50 |
| 3 or 4 portions | 1.29 (0.69) | 3.62 (0.94–14.84) | 0.06 |
| ^a^ Predictors and regression coefficients in the table are derived from the best fit model for the outcome based on the Bayesian Information Criterion (BIC). Other explanatory variables tested include household composition, caring responsibilities, smoking status, alcohol consumption, physical activity level, having long-term health conditions, amount of time spent gardening, food growing level, and having an allotment, but these were dropped in the process of improving model fit.  ^b^ Model R^2^ = 0.28 (Hosmer Lemeshow), 0.14 (Cox and Snell), 0.33 (Nagelkerke); χ^2^ (15) = 30.66  ^C^ Figures in bold are statistically significant at the 5% level (p<0.05) | | | |

**Table S6** Odds Ratios (OR) for negative (‘very negative’ or ‘somewhat negative’) as compared to neutral or positive (‘neutral’, ‘somewhat positive’ or ‘very positive’) self-reported effect of the covid-19 pandemic on participants’ diet quality, adjusted for demographic and lifestyle variables^a, b, c^

| **Variable (reference category)** | **B (SE)** | **OR (95% CI)** | ***p* value** |
| --- | --- | --- | --- |
| *Constant* | -1.40 (0.48) | 0.25 (0.09–0.61) | <0.01 |
| Gender (Female) |  |  |  |
| Male | -0.72 (0.58) | 0.49 (0.14–1.42) | 0.21 |
| Age (18–34) |  |  |  |
| 35–54 | -0.31 (0.52) | 0.73 (0.26–2.04) | 0.54 |
| 55+ | -2.56 (0.73) | **0.08 (0.02–0.29)** | <0.001 |
| Obesity (Not obese) |  |  |  |
| Obese | 1.44 (0.52) | **4.21 (1.52–11.82)** | <0.01 |
| Daily F&V intake (5+ portions) |  |  |  |
| 1 or 2 portions | 2.21 (0.77) | **9.09 (0.08–44.74)** | <0.01 |
| 3 or 4 portions | 0.73 (0.51) | 2.07 (0.75–5.68) | 0.14 |
| ^a^ Predictors and regression coefficients in the table are derived from the best fit model for the outcome based on the Bayesian Information Criterion (BIC). Other explanatory variables tested include neighbourhood deprivation, household income, household composition, caring responsibilities, higher education, smoking status, alcohol consumption, physical activity level, having long-term health conditions, amount of time spent gardening, food growing level, and having an allotment, but these were dropped in the process of improving model fit.  ^b^ Model R^2^ = 0.28 (Hosmer Lemeshow), 0.22 (Cox and Snell), 0.38 (Nagelkerke); χ^2^ (6) = 49.54  ^C^ Figures in bold are statistically significant at the 5% level (p<0.05) | | | |

**Table S7** Odds Ratios (OR) for negative (‘very negative’ or ‘somewhat negative’) as compared to neutral or positive (‘neutral’, ‘somewhat positive’ or ‘very positive’) self-reported effect of the covid-19 pandemic on physical health, adjusted for demographic and lifestyle factors^a, b, c^

| **Variable (reference category)** | **B (SE)** | **OR (95% CI)** | ***p* value** |
| --- | --- | --- | --- |
| *Constant* | 1.09 (0.64) | 2.98 (0.87–10.82) | 0.09 |
| Gender (Female) |  |  |  |
| Male | 0.50 (0.48) | 1.66 (0.65–4.24) | 0.29 |
| Age (18–34) |  |  |  |
| 35–54 | -0.60 (0.57) | 0.55 (0.17–1.67) | 0.29 |
| 55+ | -1.31 (0.66) | **0.27 (0.07–0.97)** | <0.05 |
| Higher education (No) |  |  |  |
| Yes | -1.27 (0.51) | **0.28 (0.10–0.76)** | <0.05 |
| Obesity (Not obese) |  |  |  |
| Obese | 1.46 (0.53) | **4.31 (1.54–12.69)** | <0.01 |
| Long-term conditions (No) |  |  |  |
| Yes | 0.73 (0.43) | 2.08 (0.90–4.94) | 0.09 |
| Physical activity level (Low) |  |  |  |
| Moderate | -1.95 (0.58) | **0.14 (0.04–0.41)** | <0.001 |
| High | -1.13 (0.69) | 0.32 (0.07–1.16) | 0.10 |
| Food growing level (No food grown) |  |  |  |
| 1 (very little F&V) | -1.40 (0.83) | 0.25 (0.04–1.13) | 0.09 |
| 2 | 0.15 (0.66) | 1.16 (0.32–4.26) | 0.82 |
| 3 | -0.35 (0.62) | 0.70 (0.20–2.39) | 0.57 |
| 4 | -1.57 (0.96) | 0.21 (0.02–1.18) | 0.10 |
| 5 (nearly self-sufficient in F&V) | -1.25 (0.76) | 0.29 (0.06–1.24) | 0.10 |
| ^a^ Predictors and regression coefficients in the table are derived from the best fit model for the outcome based on the Bayesian Information Criterion (BIC). Other explanatory variables tested include neighbourhood deprivation, household income, household composition, caring responsibilities, alcohol consumption, smoking status, F&V intake, amount of time spent gardening, and having an allotment, but these were dropped in the process of improving model fit.  ^b^ Model R^2^ = 0.28 (Hosmer Lemeshow), 0.28 (Cox and Snell), 0.40 (Nagelkerke); χ^2^ (13) = 60.52  ^C^ Figures in bold are statistically significant at the 5% level (p<0.05) | | | |
